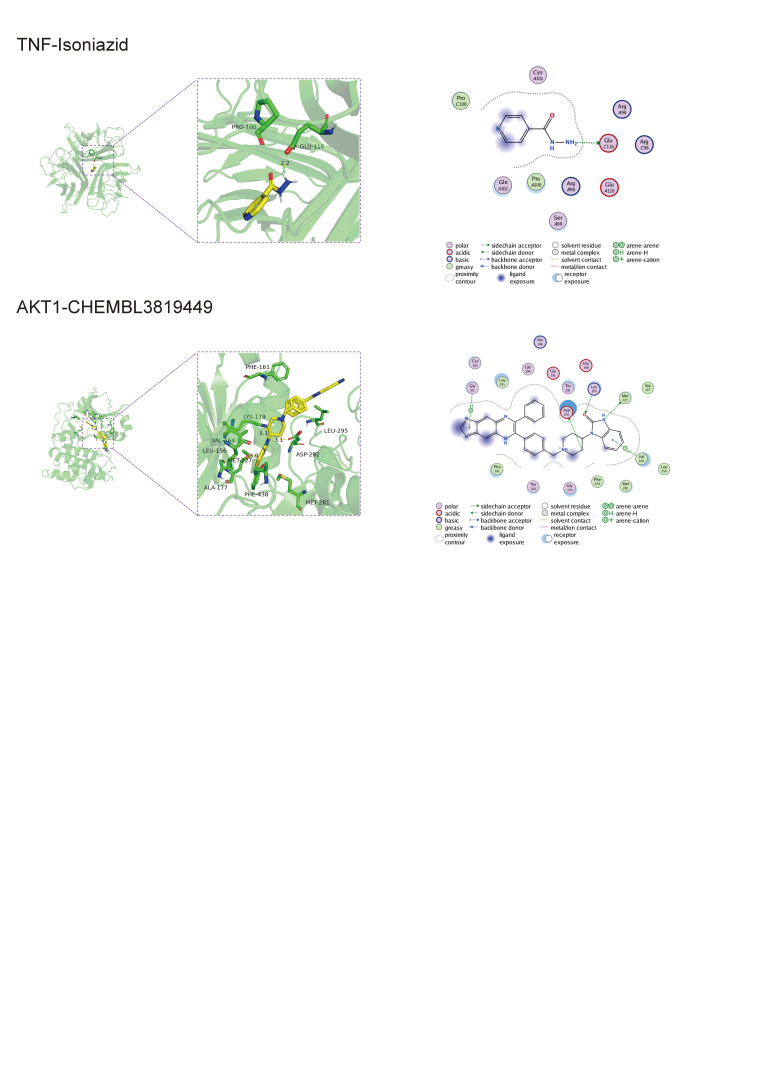# Discussion on the mechanism of Tiaoqi Xiaowei decoction in the treatment of chronic atrophic gastritis based on network pharmacology and molecular docking: An observational study

**DOI:** 10.1097/MD.0000000000038224

**Published:** 2024-05-31

**Authors:** Ruwen Yang, Jun Ouyang, Jiawei Jiang, Yuanpei Zhao, Defeng Wu, Dongmei Chen, Biao Xi

**Affiliations:** a Department of Gastroenterology, Zhenjiang Hospital of Traditional Chinese Medicine, Affiliated Hospital of Nanjing University of Chinese Medicine, Zhenjiang, Jiangsu, China.

**Keywords:** chronic atrophic gastritis, molecular docking, network pharmacology, Tiaoqi Xiaowei decoction

## Abstract

To explore the mechanism of Tiaoqi Xiaowei decoction in the treatment of chronic atrophic gastritis by network pharmacology and molecular docking. The main active components and targets of Tiaoqi Xiaowei decoction were obtained from TCMSP database. The databases of Disgenet, GeneCards, and OMIM were used to obtain chronic atrophic gastritis-related targets. The component–target–disease network was constructed by Cytoscape 3.7.1 software, and the protein–protein interaction network was constructed by String database. The core targets were screened by CytoNCA plug-in. Gene ontology analysis and Kyoto Encyclopedia of Genes and Genome pathway enrichment analysis were performed using the Metascape database. The core components and targets were subjected to molecular docking verification using AutoDock Tools 1.5.6 software, and the binding score was obtained. A total of 48 active components were identified, involving 82 action targets. Core active components such as quercetin, beta-sitosterol, kaempferol, luteolin, and naringenin, and core targets such as AKT1, TP53, VEGFA, TNF, IL6, and PTGS2 were obtained. A total of 188 signaling pathways were screened out, including cancer pathway, PI3K-Akt, IL-17, and TNF signaling pathway. Molecular docking results showed that the key components of Tiaoqi Xiaowei decoction had a favorable binding affinity with key targets. Tiaoqi Xiaowei decoction acts on multiple targets such as AKT1, TP53, VEGFA, TNF, IL6, PTGS2, and synergistically treats chronic atrophic gastritis by regulating inflammatory responses and tumor-related signaling pathways.

## 1. Introduction

Chronic atrophic gastritis (CAG) refers to a common digestive system disorder in which the gastric mucosa undergoes intrinsic glandular atrophy, reduction, or even disappearance due to various pathogenic factors.^[1]^ The definitive diagnosis primarily relies on endoscopy and histopathological biopsy. The presence of intestinal metaplasia and/or atypical hyperplasia is considered a significant precancerous lesion in the progression toward gastric cancer, with a long-term malignant transformation rate approaching 8%.^[2]^ The pathogenesis of CAG is complex and is considered to be the result of a combination of factors such as Helicobacter pylori infection, bile regurgitation, environmental factors, genetic factors, lifestyle and other factors, usually with no specific clinical manifestations.^[3]^ The general principle of CAG treatment in modern medicine is to eradicate the etiology and reduce inflammation of gastric mucosa. However, there is a lack of ideal treatment methods to effectively alleviate CAG. In recent years, traditional Chinese medicine has unique advantages in delaying or even reversing the process of gastric mucosal atrophy due to the characteristics of multicomponents, multipathways, and multi-targets.^[4]^

According to the clinical manifestations of CAG, modern scholars have classified it into the categories of “epigastric pain,” “acid reflux,” “noisy,” “epigastric fullness,” generally falling under the category of a condition characterized by deficiency in origin and excess in superficiality. Tiaoqi Xiaowei decoction is an agreement prescription based on the traditional Chinese medicine expertise of Dr Ren Nanxin in Jiangsu Province, used for the treatment of CAG. It consists of Chinese thorowax root, Szechwan Lovage Rhizome, Chinese Angelica, Finger Citron Fruit, Costusroot, Spreading Hedyotis Herb, White Paeony Root, Barbated Skullcup Herb, Villous Amomum Fruit, Nutgrass Galingale Rhizome, and Curcuma aromatica Salisb. Chinese thorowax root has the effect of soothing the liver and promoting the circulation of Qi and blood, while White Paeony Root nourishes the blood and consolidates Yin in the body. When used together, they help maintain the liver’s healthy state, ensuring that it neither disperses excessively nor lacks vital Qi. Additionally, the combination of Chinese Angelica and White Paeony Root contributes to nourishing the blood in the liver, further promoting the balance of liver Qi. Nutgrass Galingale Rhizome, Curcuma aromatica Salisb, and Szechwan Lovage Rhizome all have the ability to soothe emotions and alleviate feelings of depression. They also contribute to the improved circulation of blood in the liver. Wang Haogu, a medical expert from the Yuan Dynasty, stated that Nutgrass Galingale Rhizome is a crucial herb for addressing issues related to stagnation of Qi and blood, much like how “Materia Medica of Decoction” notes its ability to supplement Qi and blood. Curcuma aromatica Salisb and Szechwan Lovage Rhizome also have the effect of promoting the circulation of Qi and blood, as well as relieving blood stasis. “Essentials of Materia Medica” describes Curcuma aromatica Salisb ability to invigorate blood and disperse stasis, and at the same time, it can cool the heart and soothe liver stagnation. In the “Compendium of Materia Medica,” it is stated that Szechwan Lovage Rhizome is an herb beneficial for replenishing Qi in the blood. It can help alleviate tension and discomfort in the liver. Therefore, it is a suitable choice for those with deficient blood. Its pungent nature is effective in dispersing stagnation, making it also suitable for individuals with Qi stagnation. Finger Citron Fruit and Costusroot both have the function of soothing Qi and regulating breath. Costusroot has a pungent taste and can help elevate Qi, making it particularly suitable for situations where Qi stagnation hinders smooth circulation. “Seeking Truth in Materia Medica”: The herb “Costusroot” facilitates downward movement of qi and relaxes the middle region. It is considered a crucial remedy for regulating the qi distribution across the Triple Burner. Yet, among the Triple Burner, the middle is considered the most crucial. Compendium of Materia Medica: Costusroot, is a medicinal herb that regulates the Qi of the Triple Burner, capable of promoting the ascent and descent of various Qi. When the central Qi does not circulate, it is all related to the spleen. Hence, it is suitable for cases of Qi stagnation in the middle burner. In the “Compendium of Materia Medica,” it is stated that Costusroot, when paired with tonifying medicines, serves to nourish; however, when combined with purgative medicines, it acts to facilitate elimination. Finger Citron Fruit has a mild taste and belongs to the category of pungent and bitter herbs, without any drying or overly stimulating side effects. Its medicinal properties are very gentle, making it suitable for addressing various situations of Qi stagnation. While it may be slightly less effective at dispersing liver Qi compared to Immature Tangerine Fruit, and not as potent in phlegm dispersion as Tangerine Peel, it is nevertheless a valuable herb for regulating Qi stagnation in the lung, spleen, and liver meridians. Additionally, it is extremely gentle, without any excessive drying or stimulating effects, which is its main advantage. In “Convenient Reader on Materia Medica,” it is stated that Finger Citron Fruit has the effect of relieving Qi stagnation and aiding digestion, making it suitable for cases of liver and spleen Qi stagnation. Spreading Hedyotis Herb and Barbated Skullcup Herb have the effect of clearing heat and detoxifying. Modern pharmacological studies have also shown their anti-inflammatory and antitumor properties, which can be used to eliminate accumulated toxins in the stomach. Villous Amomum Fruit promotes Qi circulation, balances gastrointestinal function, and invigorates the spleen and stomach. “Transforming the Significance of Medicinal Substances” describes the effectiveness of Villous Amomum Fruit: it has a slightly spicy taste with a hint of bitterness, and its characteristic is that its aroma is quite strong. Its main function is to disperse blockages, promote smooth flow, and facilitate downward movement of qi. Its fragrance can harmonize the functions of the internal organs and flow through various meridians in the body along with other medicines. When multiple medicines are used together, in addition to regulating Qi circulation, the importance of regulating blood should not be overlooked. Together, they work to promote smooth Qi circulation, invigorate the liver, and promote blood circulation to resolve stasis. In the treatment of chronic gastritis, Villous Amomum Fruit has shown good clinical efficacy, but its specific mechanism requires further research for clarification.

Therefore, in this study, the chemical component–target–disease network of drugs was constructed based on network pharmacology, to predict and analyze the action targets and related pathways of Tiaoqi Xiaowei decoction in the treatment of CAG from the overall perspective, and to conduct molecular docking of the screened active components and target proteins, in order to provide a theoretical basis for in-depth study of the material basis and potential mechanism of Chinese herbal compound in the treatment of CAG.

## 2. Materials and methods

### 2.1. Screening of component targets of Chinese herbal compound

The chemical components of Chinese herbal compounds were obtained by TCMSP database (https://tcmspw.com/tcmspsearch.php). Through oral bioavailability (OB) and drug-likeness property (DL), the active components that simultaneously met the requirements of OB ≥ 30% and DL ≥ 0.18 were screened out. The action targets corresponding to the active components were obtained from the TCMSP database, and the results were imported into the UniProt Database platform (http://www.Unitprot.org/) for transformation into a unified gene name with the source of the qualified gene defined as “*Homo sapiens*.” The Cytoscape 3.2.1 software was used to construct the active component–target map of Chinese herbal compounds.

### 2.2. Screening of disease targets

The target information related to CAG was searched using the keywords “chronic atrophic gastritis” in the DisGeNET (http://www.disgenet.org/web/DisGeNET), GeneCards (https://www.genecards.org/), and OMIM databases (https://www.omim.org/). In the GeneCards database, a filter condition of Score ≥ 6.90 was applied. The obtained CAG-related target information from the 3 databases was merged, duplicate values were removed, and the gene names were normalized and standardized.

### 2.3. Component–target–disease network construction

The intersection of targets between the disease and Chinese herbal compound was obtained using the online Venn diagram tool, Venny 2.1.0 (https://bioinfogp.cnb.csic.es/tools/venny). The common targets and active components were then imported into Cytoscape 3.2.1 software to construct a network diagram of active components, targets, and diseases.

### 2.4. PPI network construction

The potential target proteins filtered by Cytoscape were imported into the STRING network platform (https://string-db.org/). The protein species were set to “*H sapiens*,” with the highest confidence > 0.9. All other parameters were kept at their default settings to construct the PPI network. Protein–protein interaction.tsv file was imported into Cytoscape version 3.2.1 software. The Network Analyzer plug-in in Cytoscape was employed to perform topological analysis on the network. Potential core targets were identified based on degree centrality (DC) and betweenness centrality (BC) values.

### 2.5. GO and KEGG pathway enrichment analysis

The Metascape platform was employed for conducting GO enrichment and KEGG pathway enrichment analyses on the core target genes. The GO enrichment analysis primarily encompassed biological process (BP), molecular function (MF), and cellular component (CC). The top 10 ranked data results for MF, BP, and CC were visualized using Origin 2018 software. Additionally, a KEGG bubble chart was generated using the bioinformatics platform (http://www.bioinformatics.com.cn/)..

### 2.6. Molecular docking

The core active components from the drug component–target–disease network were subjected to molecular docking with potential core targets in the PPI network. The 3-dimensional structures of the core target proteins were obtained from the Protein Data Bank archive (http://www.rcsb.org/) in PDB format. The PDB ID are shown as below: IL6: 1ALU, TNF: 2E7A, AKT1: 6NPZ, PTGS2: 5F19, TP53: 4AGP, VEGFA: 4KZN. Crystal water and original ligands were removed using Pymol 2.3.0. The protein structure was imported into AutoDockTools (v1.5.6) for hydrogenation, charge calculation, charge assignment, and specification of atom types. The POCASA 1.1 was applied to predict protein binding sites and AutoDock Vina 1.1.2 was applied to perform the molecular docking. For IL6, the parameters were set as follows: center_x = 14.5, center_y = −33.0, center_z = 0.4; for TNF were set as: center_x = −8.1, center_y = 9.6, center_z = 7.5. For AKT1 were set: center_x = −32.3, center_y = 1.2, center_z = 19.3. For PTGS2 were set as: center_x = 32.5, center_y = 28.5, center_z = 61.5. For TP53 were set as: center_x = 91.8, center_y = 91.9, center_z = −45.4. Lastly, for VEGFA, the parameters were set as: center_x = 10.4, center_y = −4.1, center_z = 22.3. The search space for all targets was set: size_x: 60, size_y: 60, size_z: 60 (with a grid spacing of 0.375 Å per grid point), and exhaustiveness was set as 10, while other parameters were set as default. Solvent molecules and ligands were removed using PyMol 2.4.0 software. Hydrogenation and electron addition operations were performed using AutoDockTools 1.5.6 software. The SDF format files of the top 5 active compounds based on Degree in the drug component–target–disease network were downloaded from the PubChem database (https://pubchem.ncbi.nlm.nih.gov/). Molecular docking calculations were conducted using the Surflex-Dock function in Sybyl-X 2.1.1 software. The quality of the docking results was evaluated based on the Total score value. The visualization of the docking results was analyzed using the molecular 3-dimensional structure display software PyMol.

## 3. Results

### 3.1. Active components and targets of TCM

**Figure 1.**
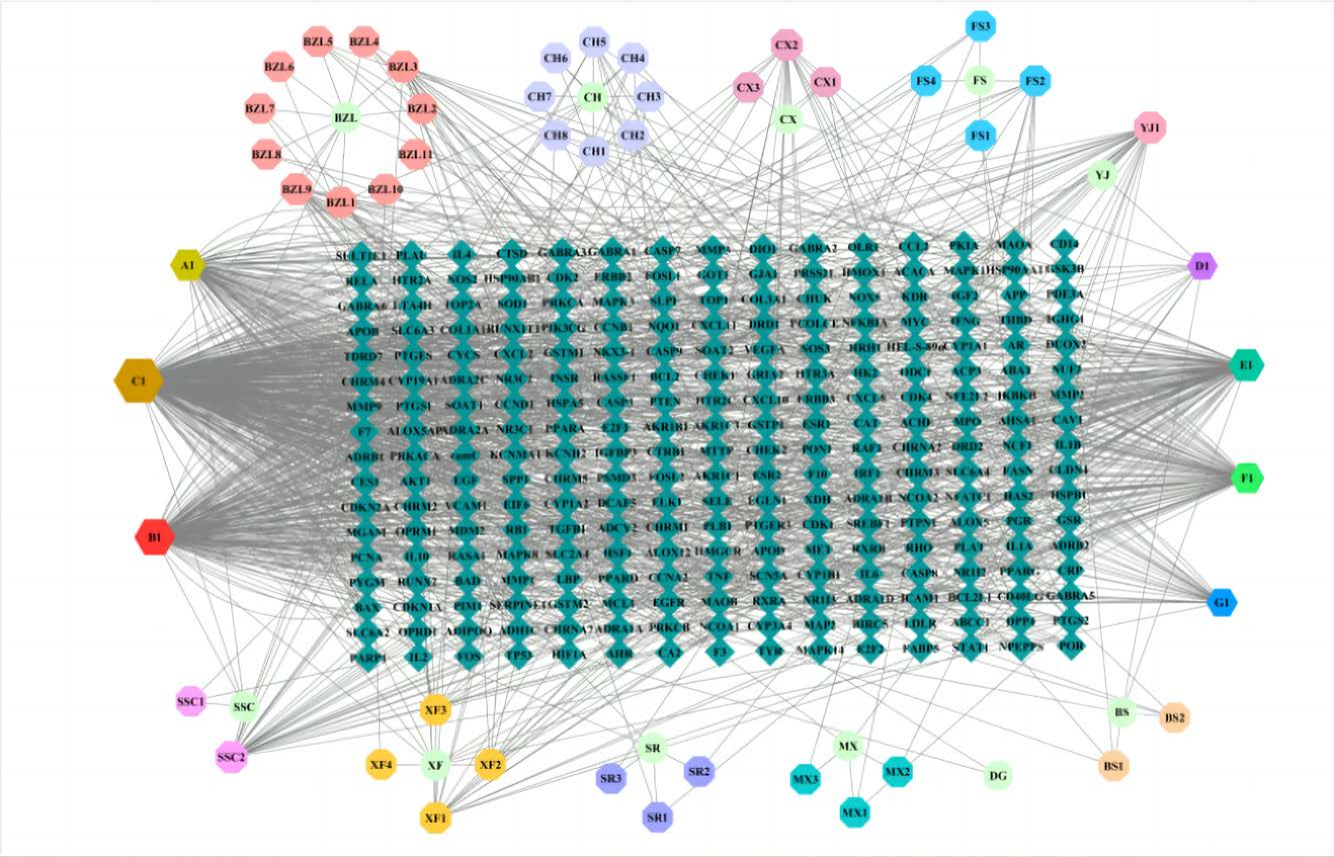
“Chinese herbal compound–active component–target” network of Tiaoqi Xiaowei decoction in the treatment of chronic atrophic gastritis.

In the TCMSP database, a search was conducted for the active components and their related targets in the 11-herb formula “Tiaoqi Xiaowei decoction.” Compounds were filtered based on the criteria of OB and drug-likeness (DL), and we selected out the active compounds that satisfied both OB ≥ 30% and DL ≥ 0.18 (Figure 1, Supplemental Digital Content, http://links.lww.com/MD/M552). This yielded 101 unique chemical components, including 17 from Chinese thorowax root, 7 from Szechwan Lovage Rhizome, 2 from Chinese Angelica, 5 from Finger Citron Fruit, 6 from Costusroot, 7 from Spreading Hedyotis Herb, 13 from BaiShao (White Paeony Root), 29 from Barbated Skullcup Herb, 10 from Villous Amomum Fruit, 18 from Nutgrass Galingale Rhizome, and 15 from Curcuma aromatica Salisb. After removing duplicates, a total of 101 unique chemical compounds were obtained (Table 1, Supplemental Digital Content, http://links.lww.com/MD/M554). These compounds were associated with 255 potential target proteins. The network of “Chinese herbal compound–active component–target” was constructed by Cytoscape 3.2. 1 software (Fig. 1).

### 3.2. Disease target screening (GeneCards disgenet OMIM)

A total of 409 related targets were found under the condition of Score ≥ 6.90 in the Genecards database. A total of 86 related targets were found in the OMIM database. A total of 203 CAG-related targets were found in the Disgenet database by retrieval, and 603 CAG-related targets were obtained after the duplicate values were removed from the 3 databases combined.

### 3.3. Active component–target–disease network construction

**Figure 2.**
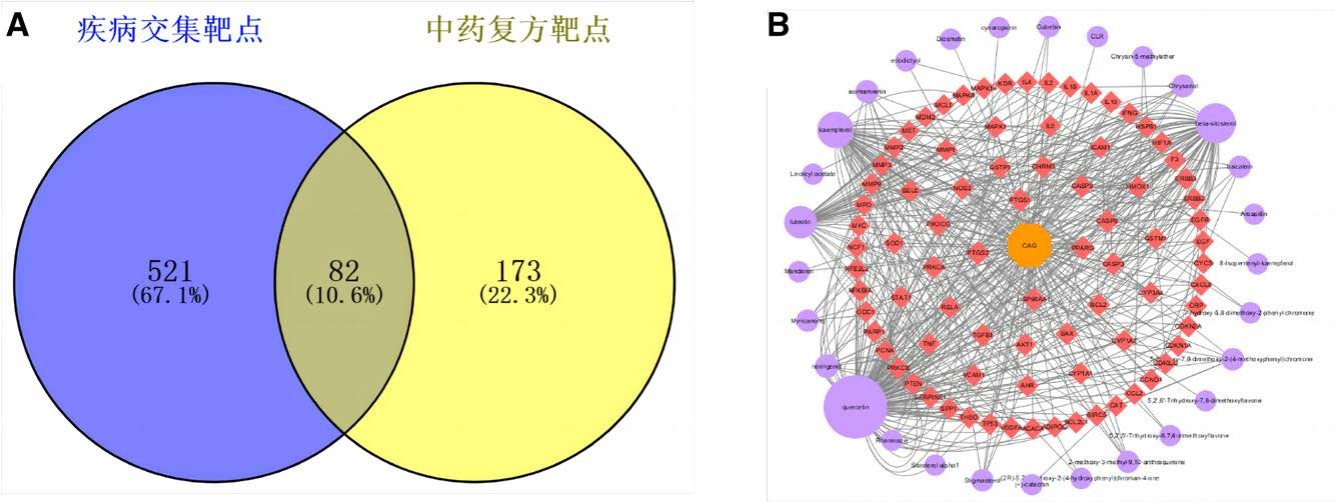
(A) Venn diagram of overlapping targets between disease-related targets and targets of Chinese herbal medicine. (B) Network diagram of “active components–target–diseases.”

The intersection of disease-related genes and the target genes of the active components was imported into the Venn diagram online drawing tool, resulting in 82 common targets between diseases and TCM (Fig. 2A). The shared targets and active components were imported into Cytoscape 3.2.1 software to construct the “Active Components–Target Proteins–Diseases” network (Fig. 2B). This network consisted of 112 nodes and 478 edges. The 112 nodes represented the 101 active components of the 11 Chinese herbs acting on 82 common targets. Each edge represented the interaction between a single herb or active components and the common targets. Node size was determined based on degree value, where larger nodes indicated more connections with other nodes. The top 5 active components, ranked by degree, were quercetin, beta-sitosterol, kaempferol, luteolin, and Naringenin.

### 3.4. PPI network construction and topology analysis

**Figure 3.**
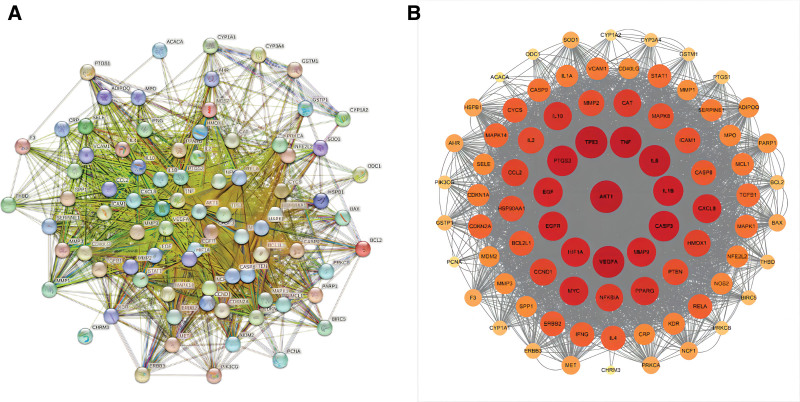
PPI network of Tiaoqi Xiaowei decoction in the treatment of chronic atrophic gastritis. PPI = protein–protein interaction network.

The 82 intersecting targets were uploaded to the STRING database to construct the PPI network (Fig. 3). Cytoscape 3.2.1 software was used for visualization and topological network analysis. The network diagram comprised 82 nodes and 3388 edges. Network Analyzer plug-in in Cytoscape was utilized for topological analysis. Nodes with higher Degrees were represented in darker colors and larger shapes (Fig. 3A and B). Simultaneously, targets were filtered based on median values of Degree and Betweenness Centrality. In each round of filtering, only targets with both values greater than the median were retained. After 3 rounds of filtering, a total of 6 potential core targets were identified, namely AKT1, TP53, VEGFA, TNF, IL6, and PTGS2.

### 3.5. GO analysis and KEGG pathway enrichment

**Figure 4.**
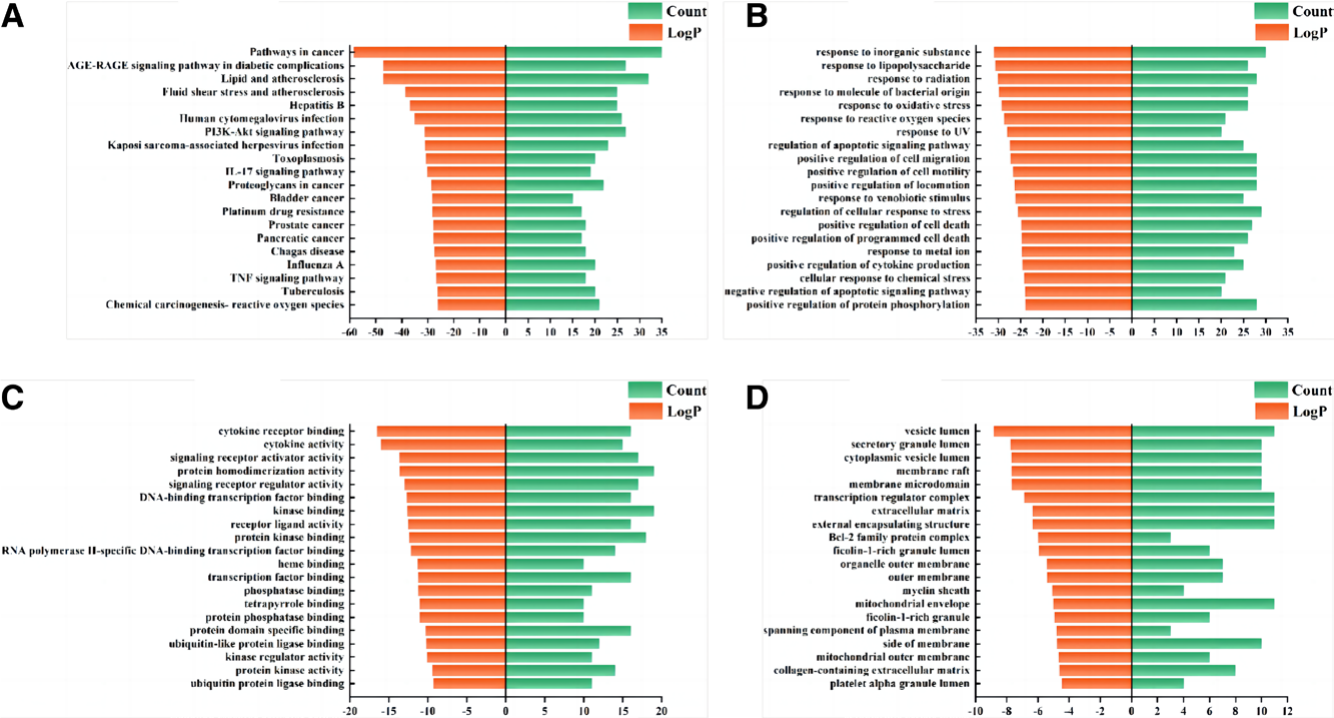
(A) KEGG pathway; (B) GO biological process; (C) GO molecular function; (D) GO cellular components. GO = gene ontology, KEGG = Kyoto Encyclopedia of Genes and Genome.

**Figure 5.**
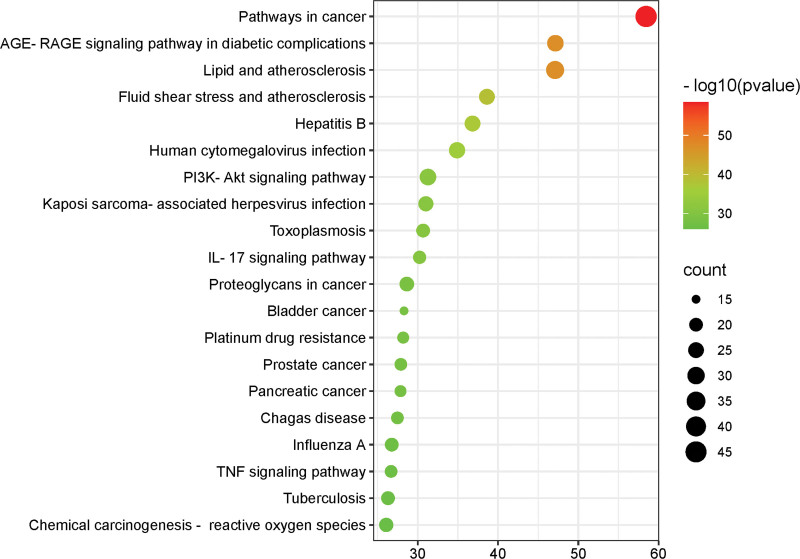
KEGG signaling pathway enrichment analysis. KEGG = Kyoto Encyclopedia of Genes and Genome.

The 82 intersecting targets were inputted into the Metascape platform for conducting GO enrichment analysis and KEGG pathway enrichment analysis. The top 10 data in terms of MF, BP, and CC were visualized and analyzed using Origin 2018 software (Fig. 4). Additionally, KEGG pathway enrichment results were visualized in a bubble plot using a bioinformatics platform (Fig. 5). The main biological processes involved in Tiaoqi Xiaowei decoction include response to inorganic substance, response to lipopolysaccharide, response to oxidative stress, response to reactive oxygen species, and regulation of apoptotic signaling pathway; molecular functions encompass cytokine receptor binding, cytokine activity, signaling receptor activator activity, protein homodimerization activity, and signaling receptor regulator activity; KEGG pathway enrichment analysis primarily involved pathways in cancer, AGE-RAGE signaling pathway in diabetic complications, PI3K-Akt signaling pathway, IL-17 signaling pathway, proteoglycans in cancer, bladder cancer, prostate cancer, pancreatic cancer, and TNF signaling pathway.

### 3.6. Molecular docking

**Figure 6.**
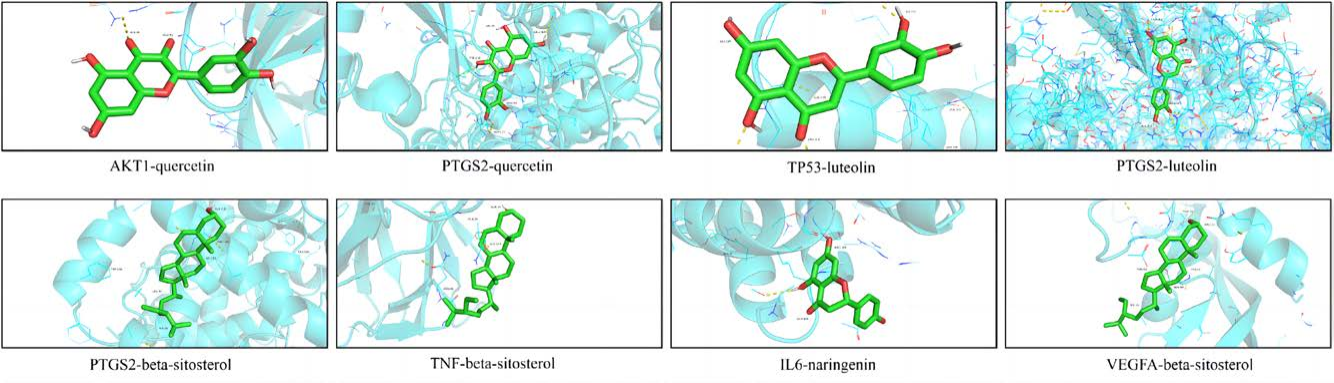
Interaction of compounds with target proteins.

**Figure 7.**
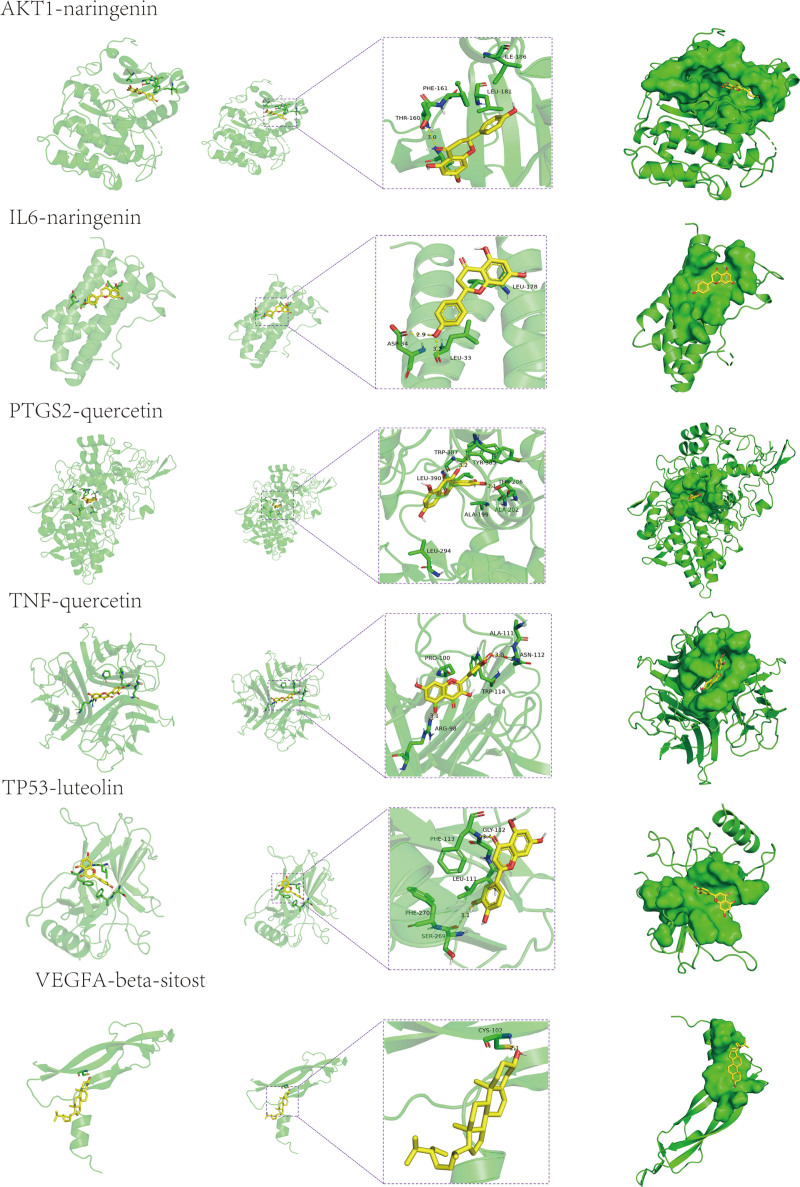
The docking results of the compound to its target with the lowest energy values.

The molecular docking calculations were performed using the Surflex-Dock software. Higher docking scores indicate better binding activity between the active components of Chinese herbal medicine and the targets. AKT1, TP53, VEGFA, TNF, IL6, and PTGS2 were selected as target genes. Small molecules, including quercetin, beta-sitosterol, kaempferol, luteolin, and naringenin, were used for molecular docking. The results of the molecular docking and the energy value were organized and presented in Table 1 and Table 2, respectively. The binding activity of each component with the targets was assessed based on the docking scores. All 5 components demonstrated spontaneous binding with the core targets. Among them, quercetin and beta-sitosterol exhibited particularly favorable binding activity with the crucial target for treating CAG. The docking results were visualized and analyzed using PyMol, and some representative binding modes are shown in Figure 6 while the results of the energy values are shown in Figure 7. Naringenin could mainly bind to the protein kinase domain of AKT1, and naringenin could interact with the unstructured region of IL6. Quercetin was able to bind with the extracellular domain od TNF, and luteolin interacted with the protein–protein interacting domain of TP53. Beta-sitosterol interacted with VEGFA via its disorder region. According to the result, the Hydrogen bond and hydrophobic interactions played the primary role in the interaction between compounds and target proteins. Naringenin formed a hydrogen bond with the THR-160 residue of AKT1, with a bond length of 3.0 Å. Additionally, it also had hydrophobic interactions with 3 residues of AKT1, which were PHE-161, LEU-181, and ILE-186. Naringenin interacted with IL6 mainly through the hydrogen bonds and hydrophobic interactions, where it formed hydrogen bonds with Il18, ASP-34, and LEU-33, and hydrophobic interactions with LEU-178. Quercetin formed hydrogen bonds with PTGS2 at THR-206 and TYR-385 residues, and it also exhibited hydrophobic interactions with LEU-294, ALA-199, ALA-202, TRP-387, and LEU-390 of PTGS2. Quercetin formed hydrogen bonds with the ASN-112 and ARG-98 residues of TNF, and also formed hydrophobic interactions with TRP-114, ALA-111, and PRO-100 residues. Similarly, luteolin formed hydrogen bonds with the SER-269 and GLY-112 residues of TP53, and hydrophobic interactions with PHE-270, LEU-111, and PHE-113. Beta-sitosterol interacted with VEGFA primarily through the formation of hydrogen bonds. It formed a hydrogen bond with the CYS-107 residue, with a bond length of 2.1 Å (Fig. 7). To validate the reliability of our docking results, we also compared the binding model of AKT1 with its known inhibitor CHEMBL3819449 and TNF with its known inhibitor Isoniazid, with Naringenin and quercetin in our finding. The data showed that their binding sites are almost the same, indicating that our docking results are relatively convincing (Figure 2, Supplemental Digital Content, http://links.lww.com/MD/M553).

**Table 1:** Molecular docking results.

Active component	AKT1	TP53	VEGFA	TNF	IL6	PTGS2
Quercetin	3.3912	3.2639	3.8505	2.7455	6.1042	7.3055
Beta-sitosterol	3.1704	3.2771	4.8709	3.6905	5.3941	6.0278
Kaempferol	1.8619	3.8856	4.4948	2.3908	4.1424	5.2814
Luteolin	2.4253	4.2647	3.5855	3.1083	4.8912	6.9599
Naringenin	2.5808	2.7223	3.1222	2.5661	5.9372	4.7073

**Table 2:** Molecular docking energy values.

Active component	AKT1 (kcal/mol)	TP53 (kcal/mol)	VEGFA (kcal/mol)	TNF (kcal/mol)	IL6 (kcal/mol)	PTGS2 (kcal/mol)
Quercetin	−7.8	−5.8	−6.4	−6.1	−6.1	−6.8
Beta-sitosterol	−7.9	−6.5	−5.3	−8.8	−6.1	−7.3
Kaempferol	−7.9	−7.2	−5.8	−6.8	−6.5	−8.2
Luteolin	−8.4	−6.6	−5.8	−8.8	−6.6	−7.6
Naringenin	−7.7	−6.4	−5.8	−8.9	−6.5	−8.8

## 4. Discussion

This study identified 48 components (Table 2, Supplemental Digital Content, http://links.lww.com/MD/M555.) and 82 targets of Tiaoqi Xiaowei decoction in the treatment of CAG. The network of “active component–target–disease” of Tiaoqi Xiaowei decoction in treating CAG was constructed. Through topological analysis, key components such as quercetin, beta-sitosterol, kaempferol, luteolin, and naringenin were screened out according to the degree value, which was mostly related to anti-inflammation, antioxidation, and antitumor.^[5]^ Studies have shown that Tiaoqi Xiaowei decoction regulates cancer pathway, PI3K-Akt signaling pathway, interleukin-17 signaling pathway, cancer signaling pathway, tumor necrosis factor signaling pathway, and other pathways by acting on core targets such as AKT1, TP53, VEGFA, TNF, IL6, and PTGS2. This subsequently influences cellular responses to lipopolysaccharides, oxidative stress reactions, and the regulation of apoptosis signaling pathways, among other life processes. Through analysis, these effects are predominantly concentrated in pathways related to inflammation and the immune system. Ultimately, molecular docking confirmed that some key components could spontaneously bind to some key targets.

Naringenin is a polyphenol compound with anti-inflammatory, immunomodulatory and anticancer effects. Beta-sitosterol is a type of plant sterol compound that plays a crucial role in the prevention and treatment of tumors.^[6]^ It can inhibit the proliferation and differentiation of tumor cells and induce the apoptosis.^[7]^ Quercetin, kaempferol, and luteolin are all flavonoids compounds, which exhibit various pharmacological activities such as anti-inflammatory, antioxidant, antitumor, and antiangiogenic effects by modulating various cellular signaling mechanisms and inhibiting the activity of carcinogenic kinases.^[8]^ Quercetin can significantly inhibit the release of inflammatory mediators and reduce the level of inflammation. At the same time, it has antitumor activity on many kinds of human tumor cells, including promoting apoptosis of tumor cells, inhibiting the growth, proliferation, metastasis, and invasion of tumor cells.^[9]^ Kaempferol can inhibit the cell cycle of various tumor cells, induce the apoptosis of tumor cells and inhibit the invasion and metastasis of tumor cells/tissues.^[10]^ Luteolin possesses significant antioxidant, anti-inflammatory, and antibacterial properties.^[11]^ Studies have also indicated that luteolin can promote tumor cell autophagy, inhibit tumor cell proliferation, and hinder migration.^[12]^

By constructing a PPI network based on potential target genes, core targets such as AKT1, TP53, VEGFA, TNF, IL6, and PTGS2 were screened. AKT can participate in mediating the occurrence and development of tumors through multiple pathways, such as promoting cell proliferation and inhibiting apoptosis.^[13]^ Inhibiting the phosphorylation level of AKT can significantly improve gastric mucosal lesions in chronic atrophic gastritis.^[14]^ p53 is a tumor suppressor gene that plays a crucial role in regulating cell differentiation, modulating the cell cycle, activating DNA repair proteins, clearing free radicals, and inducing cell apoptosis.^[15]^ It has been shown that activation and upregulation of p53 level can significantly inhibit the proliferation and migration of gastric cancer cells, promote cell cycle arrest, and then play an antitumor role.^[16,17]^ VEGFA can stimulate the proliferation and migration of vascular endothelium and induce tumor angiogenesis, which is closely related to tumor growth and metastasis.^[18]^ The research has confirmed a positive correlation between the serum expression levels of VEGFA and the degree of atrophy and intestinalization in CAG, which promotes the development of early gastric cancer.^[19,20]^ TNF is a group of cytokines that can induce cell apoptosis.^[21]^ TNF can activate inflammatory cells in the gastric mucosa to release inflammatory factors, promote gastric mucosal inflammation, and accelerate the atrophic intestinalization of the gastric mucosa.^[22]^ IL6 is an inflammatory cytokine that plays a crucial role in immune responses, acute-phase reactions, and hematopoietic regulation, which is closely associated with the occurrence, development, prognosis, and treatment of tumors.^[23,24]^ Downregulating the expression of IL6 or blocking the IL6 signaling pathway can inhibit the carcinogenesis process.^[25,26]^ PTGS2 is induced by pro-inflammatory cytokines at the site of inflammation, and is responsible for participating in the prostaglandin biosynthesis involved in inflammation and mitosis.^[27]^ Enhanced PTGS2 expression and activity can stimulate cancer cell proliferation, inhibit apoptosis, and promote angiogenesis.^[28]^

By analyzing GO entries, it can be observed that Tiaoqi Xiaowei decoction exhibits specific effects in areas such as response to inorganic substances, cellular response to lipopolysaccharides, oxidative stress response, response to reactive oxygen species, and regulation of apoptotic signaling pathways. Through KEGG analysis, Tiaoqi Xiaowei decoction is mainly concentrated in inflammation and tumor pathways, such as cancer pathway, PI3K-Akt signaling pathway, interleukin-17 signaling pathway, cancer signaling pathway, bladder cancer, prostate cancer, pancreatic cancer, tumor necrosis factor signaling pathway, and so on. The process of CAG carcinogenesis follows the classic Correa model^[29]^: normal gastric mucosa → superficial gastritis → chronic atrophic gastritis → intestinal metaplasia → dysplasia → gastric cancer. Under prolonged infiltration of various inflammatory factors, the normal gastric mucosa generates an inflammatory immune response, releasing multiple tumor immune cell factors, disrupting internal stability, creating an inflammatory and tumor microenvironment, causing genetic mutations, inhibiting apoptosis, promoting angiogenesis, and cell proliferation. This drives gastric mucosal atrophy, intestinal metaplasia, dysplasia/epithelial neoplasia, ultimately leading to carcinogenesis.^[30]^ Tiaoqi Xiaowei decoction exerts a certain intervention effect on the “inflammation-cancer transformation” process in chronic atrophic gastritis through multiple inflammatory and tumor-related pathways.

In summary, this study, based on network pharmacology and molecular docking, demonstrates that the Tiaoqi Xiaowei decoction can modulate a molecular network centered around core targets such as AKT1, TP53, VEGFA, TNF, IL6, PTGS2. This decoction exerts its therapeutic effects in the treatment of CAG by participating in the regulation of inflammatory responses and tumor-related signaling pathways. This provides new insights and directions for further experimental research in the future.

## Acknowledgments

We would like to acknowledge the reviewers for their helpful comments on this paper.

## Author contributions

**Conceptualization:** Ruwen Yang, Yuanpei Zhao, Dongmei Chen.

**Data curation:** Ruwen Yang, Jun Ouyang, Dongmei Chen.

**Formal analysis:** Ruwen Yang, Jiawei Jiang, Defeng Wu, Biao Xi.

**Methodology:** Ruwen Yang, Jun Ouyang, Yuanpei Zhao, Biao Xi.

**Validation:** Ruwen Yang, Jiawei Jiang.

**Writing – original draft:** Ruwen Yang.

**Writing – review & editing:** Jun Ouyang, Jiawei Jiang, Yuanpei Zhao, Defeng Wu, Dongmei Chen, Biao Xi.

## Supplementary Material





**Figure:**
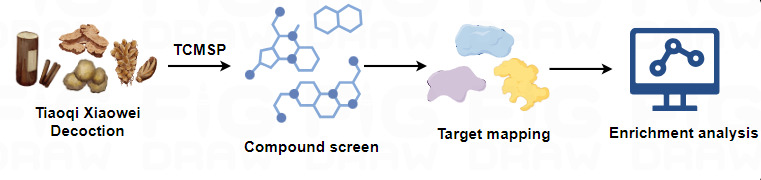


**Figure:**